# Telomere Homeostasis: Interplay with Magnesium

**DOI:** 10.3390/ijms19010157

**Published:** 2018-01-05

**Authors:** Donogh Maguire, Ognian Neytchev, Dinesh Talwar, Donald McMillan, Paul G. Shiels

**Affiliations:** 1 Emergency Medicine Department, Glasgow Royal Infirmary, Glasgow G4 0SF, UK; Donogh.Maguire@glasgow.ac.uk; 2 Academic Unit of Surgery, School of Medicine, University of Glasgow, Glasgow Royal Infirmary, Glasgow G4 0SF, UK; Donald.McMillan@glasgow.ac.uk; 3 Section of Epigenetics, Institute of Cancer Sciences, University of Glasgow, Glasgow G61 1QH, UK; o.neytchev.1@research.gla.ac.uk; 4 The Scottish Trace Element and Micronutrient Reference Laboratory, Department of Biochemistry, Royal Infirmary, Glasgow G31 2ER, UK; Dinesh.Talwar@ggc.scot.nhs.uk

**Keywords:** telomeres, telomerase, TERT, mitochondria, oxidative stress, magnesium

## Abstract

Telomere biology, a key component of the hallmarks of ageing, offers insight into dysregulation of normative ageing processes that accompany age-related diseases such as cancer. Telomere homeostasis is tightly linked to cellular metabolism, and in particular with mitochondrial physiology, which is also diminished during cellular senescence and normative physiological ageing. Inherent in the biochemistry of these processes is the role of magnesium, one of the main cellular ions and an essential cofactor in all reactions that use ATP. Magnesium plays an important role in many of the processes involved in regulating telomere structure, integrity and function. This review explores the mechanisms that maintain telomere structure and function, their influence on circadian rhythms and their impact on health and age-related disease. The pervasive role of magnesium in telomere homeostasis is also highlighted.

## 1. Introduction

Ageing is not a simple passive degenerative process, but one regulated by distinct biochemical pathways. It has been classified by a number of common hallmarks that are shared across taxa. These can be sub-classified into primary, antagonistic, and integrative hallmarks, one of which is telomere attrition [1].

Telomeres are nucleo-protein complexes at the linear ends of vertebrate chromosomes containing (TTAGGG)_n_ DNA repeats, which are essential for maintaining genomic integrity [2]. Telomere attrition is an integral part of the end replication problem [3,4] and acceleration of the rate of telomeric sequence loss is a feature of a plethora of non-communicable diseases [5]. Telomere shortening has been reported to accelerate in association with physical and psychological stress exposures [5]. Critical cellular telomeric shortening can trigger a persistent DNA damage response (DDR), that signals the cell to enter senescence, or undergo apoptosis, dependent upon the relative level of genomic damage the cell has accrued during its replicative lifespan [2].

Telomere shortening, however, is not a unidirectional process: stem cells, cancer cells, as well as some differentiated cells—including B and T lymphocytes—can counter telomere shortening and maintain the length of their telomeric DNA despite mitotic activity, typically through the action of the holoenzyme telomerase [6]. Approximately 90% of human cancers demonstrate up-regulation of telomerase activity [7], while most of the remaining 10% use an alternative lengthening of telomeres (ALT) mechanism [8]. This ability to preserve telomere function is necessary to confer replicative immortality, one of the hallmarks of cancer [9]. 

Telomere maintenance is a dynamic process, which is responsive to metabolic and endocrine mediators of cellular stress [10]. Factors that mitigate the effect of cellular stress, such as free radical scavengers and antioxidants, may thereby offer the possibility of slowing the rate of telomere attrition [11].

## 2. Magnesium Homeostasis and Disease

Magnesium (Mg^2+^) is one of the most abundant metal ions in cells and plays a variety of roles, including the stabilization of DNA, RNA, adenosine triphosphate (ATP), and other nucleotide triphosphates/dioxynucleotide triphosphates (NTPs/dNTPs) [12]. It participates in over 600 enzymatic reactions, including all those that involve ATP, and is essential for maintenance of DNA structure, cellular excitability and cellular health span [13,14,15]. Mg^2+^ deficiency has been implicated in many disease processes, including type 2 diabetes (T2DM) [16], Alzheimer’s disease [17], and cancer [18,19,20,21], while Mg^2+^ supplementation has been shown to be beneficial for blood pressure control, endothelial function, insulin sensitivity and T2DM [22,23,24]. In vitro, low [Mg^2+^] has also been shown to promote traits associated with senescence in endothelial cells, including slower cell growth and up-regulation of p21 (a cell cycle inhibitor) and IL-1α (a pro-inflammatory cytokine) [25]. However, cells appear to have a remarkable ability to preserve intracellular [Mg^2+^] in spite of a low extracellular concentration of this ion [26], or a dynamic metabolism [27]. On the other hand, intracellular [Mg^2+^] increases several fold during the log phase of cell growth, in spite of a constant ATP/ADP and extracellular [Mg^2+^], providing further evidence for the hypothesis that cellular [Mg^2+^] is actively regulated [27].

In the extracellular environment, Mg^2+^ is involved in inhibiting ectopic calcification, which is a key feature of accelerated physiological ageing [28,29,30]. Mg^2+^ has been shown to interfere with the growth of ectopic calcium phosphate crystals in blood vessels [30] and to delay the morphological shift from primary to secondary calciprotein particle formation [31]. In addition, Mg^2+^ may also contribute to the osteogenic transformation of vascular smooth muscle cells [30]. Mg^2+^ has been found to correlate negatively to both Fetuin-A and FGF23 in serum [32]. Fetuin-A is a major calcification inhibitor in the circulation [31,32], while FGF23 is an endocrine factor that can contrast calcification by inducing phosphate excretion [33,34].

Mg^2+^ transport across membranes is still poorly understood, but homeostasis at an organismal level appears to be maintained by modulating absorption in the intestine and reabsorption in the kidney [30,35]. In both cases, Mg^2+^ is imported apically by TRPM6 channels, transferred paracellularly across tight junctions with the contribution of claudin-16 and claudin-19, and exported basolaterally by the SLC41A1 Na/Mg exchanger [35,36]. In all cell types, Mg^2+^ is imported by TRPM7 channels and exported by the action of SLC41A1 [35,36]. Another important member of the SLC41 family is the Na/Mg exchanger SLC41A3, which is involved in transport across the mitochondrial membrane along with the Mrs2 channel [35,36,37].

## 3. Magnesium and Telomeres

Cytosolic Mg^2+^ concentration is approximately 1 mM [30], but significant reserves are present in bound form, mainly chelated by ATP, or within intracellular compartments, especially inside the mitochondria [38]. In a manner analogous to the regulation of calcium biochemistry, release or uptake of Mg^2+^ can cause large local shifts in [Mg^2+^], which in turn can regulate a variety of processes, including the regulation of flow of metabolites through glycolysis, the TCA cycle, oxidative phosphorylation, and ATP export from the mitochondria [38]. Critically, Mg^2+^ plays a role in telomere maintenance and the activity of telomerase [39]. These interactions are discussed in context in the following sections.

### 3.1. Telomere Attrition

The main driver of telomere attrition is DNA replication. However, the amount of telomeric sequence loss can vary dramatically, from the theoretical minimum of less than 10 base pairs (bp) lost per replication, to an average of 50–200 bp, up to about one thousand bp [40]. Reactive oxygen species (ROS) and oxidative stress produced by dysfunctional mitochondria can accelerate telomere attrition [40,41], while reducing ROS can mitigate this effect [11].

Telomeric sequences display a particular sensitivity to ROS-induced damage, as they are guanine-rich (G-rich) and have stacked G repeats. G has the lowest oxidation potential among the four DNA bases [42,43], and vulnerability to oxidative stress increases exponentially when G nucleotides are stacked [44]. The human telomeric DNA repeat stretch has been shown to be more sensitive to strand breakage, particularly at the G repeat, compared to non-telomeric sequences [45,46,47,48]. Additionally, DNA repair appears to be less efficient at telomeres in comparison to the rest of the genome [41]. The shelterin protein complex, which ‘caps’ telomeres, provides protection and impairs access to nucleases [49]. This prevents telomere ends from being recognised as DNA breaks, from being subjected to nuclease activity inherent in DNA repair processes, and from acting as a trigger for apoptosis [50]. In light of these considerations, it is thought that higher levels of ROS lead to an accumulation of unrepaired DNA damage at the telomeric region, which causes a larger DNA sequence loss during subsequent replication [40,51]. As such, the stacked G nucleotides integral to telomere structure may act as a redox-sensitive ‘alarm system’ that senses and signals the presence of oxidative damage before it can cause significant damage to the rest of the genome.

**Figure 1:**
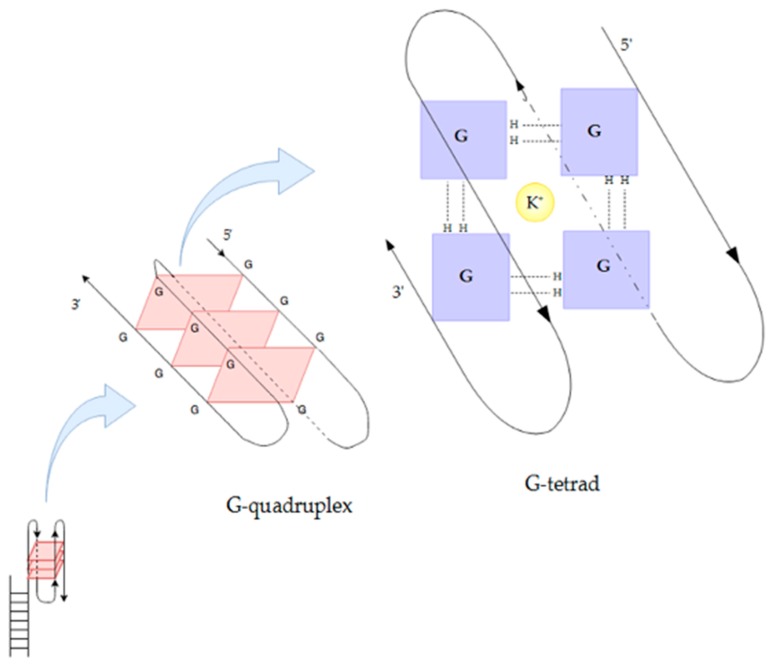
Schematic representation of an antiparallel G-quadruplex formed from a single DNA strand. This type of structure can form at telomere ends, protecting and stabilising the single strand overhang. The G-quadruplex is composed of several stacked G-tetrads, each of which is in turn formed by four guanines (G) joined by Hoogsteen hydrogen bonding and stabilised by a monovalent cation (K^+^ in this figure).

Secondary telomeric DNA structure is also important for telomere maintenance. In particular, it has been shown that the formation of G-quadruplexes prevents DNA repair machinery and telomerase from accessing the telomeric region [49,52]. G-quadruplexes can be formed by either a single DNA strand, or multiple DNA strands, when four G bases join by Hoogsteen hydrogen bonding to form a square planar structure called a G-tetrad, and two or more G-tetrads stack on top of each other in the presence of monovalent K^+^ and Na^+^ ions to form a G-quadruplex (Figure 1) [53]. G-quadruplexes may form stable nano-wires (G-wires) when guanine-rich DNA strands are annealed in the presence of suitable divalent cations [54]. Mg^2+^ can cause a configuration switch in G-quadruplex structures [55] and has been reported to stabilise G-wires [53,56]. G-wires are highly resistant to denaturation and have been reported to interfere with telomerase function [53,55,57]. In addition, some proteins appear to recognise specifically G-quadruplex structures, such as the GAR domain of the shelterin protein TRF2 [58].

Mg^2+^ has been reported to enhance the stability of G-quadruplexes at oncogene promoter regions and prevent conversion to their pro-oncogene duplex form [59] and thus can act as an off switch in transcriptional regulation [60]. For instance, the promoter of the proto-oncogene *c-Myc* contains a parallel-stranded G-quadruplex that appears to bind Mg^2+^ most avidly [53,59]. Telomere attrition was found to be faster when cells were cultured in Mg^2+^-deficient conditions [26]. It has recently been reported that telomere G-quadruplexes may render DNA particularly sensitive to direct ultra-violet radiation (UV) damage and allow the oxidation of telomeric Gs even at wavelengths that are below the G ionization potential [61]. The implications of this observation for cancer neogenesis, progression and treatment remain to be explored. Recently, however, melanoma risk among individuals with light skin has been hypothesised to be mitigated by shortening of their telomeres through polygenic adaptation [62]. This may explain, in part, the shorter telomere lengths in Caucasians than in sub-Saharan Africans [63]. G quadruplex sensitivity to UV light may thus provide a mechanistic/structural basis for telomeres within the framework of such polygenic adaptation and cancer risk.

### 3.2. Magnesium and Telomeric Structure

Telomeric chromatin structure and integrity is impacted upon by Mg^2+^ biochemistry [39]. In particular, this can involve regulation and maintenance of higher order telomeric chromatin structure. More than 50% of telomeres localise to the lamina, a scaffold of intermediate filaments called lamins, that contributes to chromatin organisation, supports the mechanical stability of the nucleus, while also modulating signal transduction and transcription regulation [64,65]. The nuclear lamina comprises two lamin types, A and B, which are organised into distinct but overlapping networks that perform different functions within the nucleus [64]. Modulation of the lamin tail domain, which is recognised by lamin-binding proteins and provides an attachment site for chromatin and other structural proteins, has been demonstrated to be dependent on the presence of Mg^2+^ [64]. Lamin B1 has been shown to be sensitive to Mg^2+^ [64]. Down-regulation of lamin B1 is required for progression to full senescence, which triggers both local and global chromatin modifications [65]. Indeed, the decrease of lamin B1 can serve as a biomarker of senescence [66].

### 3.3. The Role of Telomeric Repeat-Containing RNA (TERRA)

Regulation of higher order telomeric structure is also a general feature of the epigenetic landscape and involves non-canonical epigenetic modifiers, such as non-coding RNAs (ncRNAs). An important player in this context is TERRA, a class of long ncRNA (lncRNA) transcribed from insulated promoters within telomeric and sub-telomeric regions that largely escape heterochromatinization. This class of RNA varies considerably in length, from approximately 100 bases to 9 kilobases (kb), and contains telomeric repeats [58]. The accumulation of mutations within the sequence allows TERRA to have, at the same time, specific binding in *cis* to the telomere of origin and generalised activity in *trans* on any chromosome [67].

While the picture of TERRA’s full role inside the cell is not yet clear, evidence has been accumulating on TERRA fulfilling a variety of functions related to telomere maintenance [58,67,68]. TERRA can interact with telomeric DNA to form hybrid DNA-RNA G-quadruplexes, interact with components of the shelterin complex, and bind telomerase [58]. TERRA is also thought to play a role in organising and expanding telomeric heterochromatin [69] and to help in telomere homeostasis. Indeed, TERRA appears to be expressed at a higher rate from short telomeres, to nucleate enzyme clusters by binding to multiple telomerase copies, and to recruit them to its telomere of origin [67].

### 3.4. Telomerase Activity in the Nucleus

Apart from its contribution to the maintenance of telomere structure and function, Mg^2+^ also has implications for the regulation of telomerase activity. The telomerase holoenzyme is formed by two essential components, the telomerase RNA that provides the template sequence for reverse transcription (TERC) and the catalytic protein component, telomerase reverse transcriptase (TERT), as well as by a number of accessory proteins [70]. It preferentially elongates short telomeres, because the stability of the shelterin complex is higher on longer telomeres [71]. This canonical function of telomerase requires Mg^2+^, which has therefore the potential to modulate its activity [6,39]. As with telomere shortening, elongation is also restricted to the S phase of the cell cycle, which is the only point at which active telomerase can access and elongate telomeres [71].

Interestingly, TERT is known to be subject to alternative splicing, with more than 20 known isoforms described in man, many of which lack a functional reverse transcriptase (RT) domain [72,73]. These isoforms have been hypothesised to act as antagonists and compete in binding TERC, and to be involved in some of the non-canonical functions of TERT which appear to be largely TERC-independent and even RT-independent [74]. These are discussed below.

### 3.5. Non-Canonical TERT Functions in the Nucleus

In the nucleus, TERT can act as a transcription factor (TF) and affect genes involved in energy metabolism, ‘stemness’, and proliferation, including the up-regulation of transfer RNA (tRNA) transcription, interaction with NF-κB and β-catenin to activate their respective pathways, and regulation of cyclin D1 expression [70]. Interestingly, β-catenin has, in turn, been shown to bind to the TERT promoter, suggesting a possible feed-forward loop that supports stemness and proliferation in wnt-dependent cancers [70].

Another emerging role for TERT is as an RNA-dependent RNA polymerase [73]. Human TERT (hTERT) has been shown in cancer cells to catalyse the creation of double-stranded RNA (dsRNA) from a non-coding RNA template, which is processed as a small interfering RNA (siRNA) and induces silencing, targeting in particular transcription from centromeres and transposons and possibly helping in the maintenance of heterochromatin [73].

### 3.6. Non-Canonical TERT Functions in the Mitochondria

TERT is known to be shuttled between the nucleus, cytoplasm, and mitochondrion [10]. The localisation of TERT within different cellular sub-compartments is a responsive process, which is regulated by growth factors, oxidative stress, caloric restriction [75], as well as antigen exposure in lymphocytes [76]. During increased oxidative stress, TERT is excluded from the nucleus and translocates to the mitochondria where it is reported to exert an antioxidant effect and promote mitochondrial health and cell survival [76]. Indeed, dampening of mitochondrial ROS production correlates with mitochondrial TERT accumulation [75]. The exact role of TERT in mitochondria is not yet clear, but several possibilities have been proposed. It has been shown to increase potential across the inner mitochondrial membrane, decrease ROS production, protect mitochondrial DNA (mtDNA) from damage, inhibit intrinsic apoptosis, and up-regulate activity and efficiency of the electron transport chain (ETC) [73,74].

TERT may also be tied to the general energy metabolism of the cell by interacting with the mammalian target of rapamycin (mTOR) pathway and the activities of the sirtuin family, key mediators in the mitochondrion-telomere-ribosome biogenesis (MTR) trinity and regulators of the cellular epigenetic landscape.

### 3.7. Telomeres and mTOR

The mammalian target of rapamycin complex 1 (mTORC1) pathway plays a crucial role in integrating signals from growth factor and nutrient availability sensors, and in regulating a variety of metabolic downstream pathways, including protein synthesis, cell cycle progression, energy metabolism, mitochondrial function, autophagy, and cell survival [77]. Intriguingly, hTERT has been shown to bind to and inhibit mTORC1, leading to increased autophagy [78] and possibly mimicking other effects of caloric restriction (CR). Conversely, mTOR activation of TERT has also been proposed [79].

Notably, mTOR demonstrates high sensitivity to Mg^2+^ oscillations [80] and it is therefore possible that Mg^2+^ acts as a regulator of the balance between anabolism and catabolism [81]. The observation that serum [Mg^2+^] increases in animals undergoing hibernation, or torpor, supports this hypothesis, because this increase cannot be due to nutrition and is therefore likely to be the result of extrusion from cells [81]. It is worth mentioning that depletion of cellular Mg^2+^ reserves, with likely repercussions on the metabolic balance, has been identified as both a cause and a consequence of ageing [26,39,82]. 

## 4. Mitochondrial Health, Ageing and Cancer

Mitochondrial homeostasis has been shown to deteriorate with age [83,84], and multiple mechanisms link it to telomere attrition [11]. An adequate concentration of mitochondrial Mg^2+^ ([Mg^2+^]_mito_) is essential for maintaining the integrity of the electron transport chain, as well as for controlling the flow of metabolites through glycolysis and the TCA cycle [38]. Consequently, [Mg^2+^]_mito_ is a key determinant of cellular oxidative resilience, i.e., the ability to prevent or repair oxidative damage. This is pertinent, as mitochondrial dysfunction leads to increased ROS production, which in turn causes increased DNA damage and accelerated telomere attrition [11]. Furthermore, mitochondrial dysfunction induces specific nuclear gene expression through “retrograde signalling” [76].

On the other hand, telomere dysfunction activates p53 signalling, which represses the expression of PGC1-α/β, the master regulators of mitochondrial biogenesis [85]. In addition, a PGC1-α/β-independent pathway has been described, and its activity has been shown to decline during ageing along with changes in mitochondrial nicotinamide adenine dinucleotide (NAD^+^ levels [86]. This decline can be reversed if intramitochondrial NAD^+^ is increased by supplementation of its metabolic precursor **nicotinamide mononucleotide** (NMN) [86]. The decline in mitochondrial NAD^+^ appears to be due to an imbalance between nuclear and mitochondrial-encoded subunits, which is associated with an accumulation of HIF-1α, creating a pseudohypoxic state in spite of normal oxygen levels [86]. In mice, deletion of SIRT1 accelerates HIF-1α production, whereas raising NAD^+^ levels in old mice restores mitochondrial function to that of a young mouse in a SIRT1-dependent manner [86].

Interestingly, Mg^2+^ deficiency was shown to decrease resistance to hypoxia in vivo [87] and to impair the upregulation of HIF-1α caused by hypoxia in cell culture [88], while Mg^2+^ supplementation helps in reducing axon pathfinding errors caused by hypoxia [89] and has a neuroprotective effect in preterm birth cases [90]. It is worth noting that HIF-1α activation and pseudo-hypoxic reprogramming also occurs in cancer, shifting the cell towards a glycolytic phenotype [91]. 

It has been reported that an elevated concentration of TERT within the mitochondrion ([TERT]_mito_) may be associated with a prolonged period of reduced psychological stress and improved rest [92]. These observations are in keeping with the finding that telomere length has been shown to negatively correlate with increased cortisol responsiveness [93], anxiety and depression [94,95], lower socio-economic status [96], chronic low-grade inflammation, a higher frequency of adverse early life events [97,98], and adult daily life stressors [99]. For example, Alzheimer’s dementia (AD) and cardiovascular disease are characterised by mitochondrial dysfunction and increased ROS production [100,101], while also being associated with accelerated telomere shortening [102,103].

## 5. Telomere Length and Disease

Telomere attrition is a hallmark of ageing and age-related disease. However, it has been reported that individuals with telomeres that are longer than expected, given their age and gender, in healthy tissue (i.e., “constitutively” long telomeres) may have a higher risk for major cancers than individuals with “constitutively” shorter telomeres [104]. This is not intuitive, as established physiological stressors [105,106,107] have been reported to accelerate age-related telomere shortening. It is therefore of interest that the majority of cancerous tissues have been reported to display short telomere lengths in comparison to a previous disease-free measurement [104]. The reasons for this discrepancy remain unclear. One consideration that may be pertinent to this observation is the fact that telomerase appears to have a different activity in healthy and cancerous cells [71]. In health, telomerase targets short telomeres preferentially and extends them with high processivity, while in cancer cells telomerase appears to target telomeres at random and extend them with low processivity [71]. In combination with a significantly elevated rate of cell proliferation, this may explain why telomeres tend to be shorter in cancerous tissue, in spite of the presence of active telomerase. In addition, this leads to the preservation of average telomere length (TL), providing replicative immortality, while at the same time allowing the existence of critically short telomeres that act as a source of genomic instability (t-stumps) [71,108,109,110,111,112].

The impact of TL dynamics remains controversial in oncoscience, with equivocal findings highlighting an onco-protective role for long telomeres, while other studies indicate a tumour-promoting association for long telomeres [113,114]. A large number of meta-analyses have also reached conflicting conclusions, with some reporting short telomeres to be associated with increased cancer risk [115,116,117], and others no significant overall effect [118,119,120]. Interestingly, one meta-analysis found that, while shorter TL is associated with lower survival, a lower TL ratio of tumour versus healthy tissue was associated with better survival [121].

It is worth mentioning that these meta-analyses carry significant caveats: results were highly heterogeneous and often not statistically significant; in addition, correcting for a large number of potential confounding factors, including age, gender, stress, smoking status, duration of treatment, etc. was not possible. The difficulty in finding a clear link may reflect the fact that TL can play a variety of roles in different types and at different stages of cancer. Additionally, epigenetic and technical issues relating to telomere measurement may also confound many studies. TL, for example, is a highly heritable genetic trait that displays wide variation across the population. It is significantly influenced by parental age at the time of birth [122,123]. Issues relating to differing TL measurement methodology between labs also influence study outcomes and meta analyses and are elegantly covered by Martin-Ruiz et al. [124]. It is worth noting that most methods provide an average TL value; however, TL varies significantly between cells and even between chromosomes within the same cell, and a single uncapped telomere can trigger senescence [125,126,127]. In addition, many studies use white blood cell DNA; in such cases, reliability of the results is further limited by the fact that different white blood cell types have different average TL and their abundance in blood, both in relative and in absolute terms, can vary significantly depending on the subject and their immunological status [125,128,129]. These issues may, however, be resolved through the use of intrinsic and extrinsic epigenetic clocks [130,131,132] in future studies.

The role of Mg^2+^ in cancer prevention and genesis is also complex. Epidemiological studies have shown an inverse relationship between Mg^2+^ consumption and cancer prevalence [20,133], and in vitro studies have indicated that Mg^2+^ may be protective during the early reprogramming phase [134]. In contrast, Mg^2+^ supplementation to an established tumour has been shown to promote tumour growth [134,135,136]. Interestingly, a similar double-edged relationship, based on tumour progression, has been established for thiamine supplementation as well [137].

Any pro-growth effect of Mg^2+^ may be due to a direct DNA stabilization effect, but may also be attributable to the enhanced performance of the non-oxidative arm of the pentose phosphate pathway (PPP) through co-operative optimization of transketolase (TK) function by Mg^2+^ and thiamine [138] resulting in increased production of ribose-5-phosphate [139].

## 6. Telomeres, Metabolism, and Circadian Rhythms

The circadian rhythm relies on a transcription-translation feedback loop that regulates numerous metabolic processes during the day-night cycle and is reported to target 43% of the human protein-coding genes [140]. In addition, 56 of the top 100 most commonly prescribed medications directly target the products of circadian genes [140]. In mammals, clock pathways operate in individual cells, but are synchronised by the master clock in the suprachiasmatic nucleus (SCN) in the hypothalamus, which in turn is sensitive to light cues from the retina [141,142]. Circadian fluctuations are present in the sleep/wake cycle, body temperature, hormone production, cell proliferation and DNA repair [143]. 

TERT mRNA expression has also been reported to oscillate with the circadian rhythm [144]. In mice, deficiency of the clock gene *CLOCK* has been demonstrated to cause lower telomerase activity, loss of rhythmicity in *TERT* mRNA expression, and shorter telomeres [144]. Similarly, hospital physicians who work a regular day time pattern have regular and rhythmical circadian oscillation of telomerase activity, while physicians who work a shift pattern in the emergency department lose the normal circadian rhythms of telomerase activity [144]. This dysregulation of telomerase activity may contribute to accelerated ageing and be linked to the higher risk seen in shift workers for a variety of health conditions [145]. In addition, cellular senescence appears to impair circadian rhythms [146], so it’s tempting to speculate that the accumulation of senescent cells with age may contribute to the observed disruption of the circadian clock, creating a possible vicious cycle. Loss of circadian regulation is a feature of mammalian ageing [29].

Given its role in energy metabolism and redox homeostasis, it is perhaps not too surprising that Mg^2+^ flux across the cell membrane, especially in the SCN, was recently identified as a regulator of circadian metabolic oscillations, leading to speculation that [Mg^2+^] fluctuations may be a component of the cellular clock [80] and that Mg^2+^ deficiency may compromise sleep quality and circadian rhythm [147]. If so, this would indicate that Mg^2+^ is a key component of cellular ageing and thus physiological ageing. Furthermore, *N*-methyl-d-aspartate receptors (NMDARs) within the SCN, which orchestrate light-induced phase resetting of the circadian rhythm [148], are Mg^2+^-dependent [149]. An indirect effect of cellular Mg^2+^ homeostasis that may be mediated through hepatocyte activation of cortisone remains relatively unexplored from a circadian perspective [150,151]. The increased cortisol activity may cause further Mg^2+^ loss and aggravate the circadian disruption [152].

Taken together, these findings indicate that the circadian regulation of telomerase activity and Mg^2+^ homeostasis may play an important role in health, disease, and ageing [144,147]. In light of the twin role of telomerase and Mg^2+^ in telomere maintenance and oxidative resilience, circadian disruption could impair the homeostasis and synchronization between telomerase activity and localisation, local [Mg^2+^], DNA replication, and redox balance. This could in turn cause accelerated telomere attrition, increased ROS production, and faster ageing.

## 7. Conclusions

**Figure 2:**
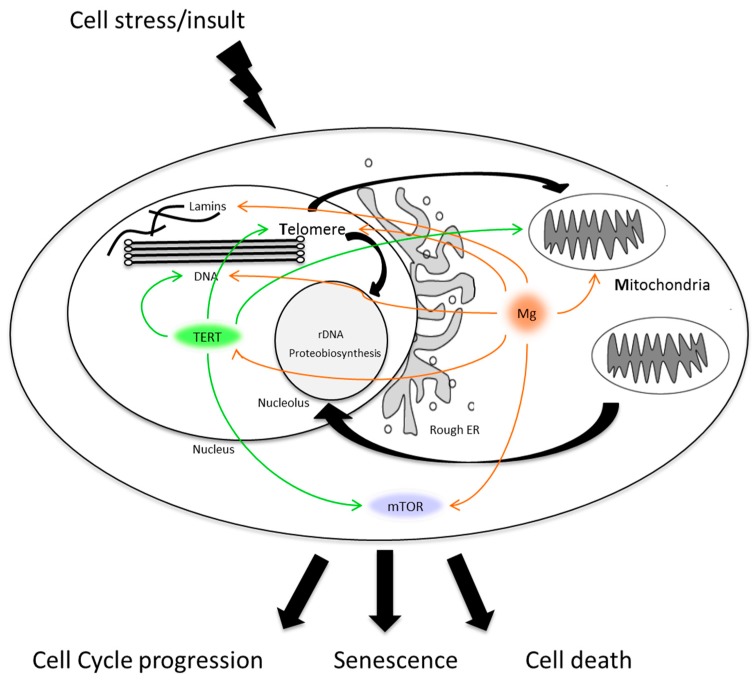
A schematic of the interplay between mitochondria, telomere nucleo-protein complexes and proteo-biosynthesis regulation in response to cellular stress/damage. In response to the stress, the cell will decide how much fuel to burn and energy to expend to repair any damage accrued. If the cell can repair the damage it can progress through the cell cycle. Too much damage and the high risk of onconeogenesis induces cell death. Senescence, strictly in this simplified scenario, sees the cell carry accumulated, sub-lethal damage, as wear and tear, leaving the cell metabolically active, but physiologically non-contributory to the function of the tissue and organ in which it resides. The pervasive and overlapping roles that TERT (green) and magnesium (orange) play in targeting major components of the MRT trinity are highlighted. ER, mTOR/.

Telomere structure and function are tightly linked to the homeostatic regulation of cellular metabolism [153]. Cellular energy production, energy expenditure and protein biosynthesis are coupled under the aegis of the MTR (Figure 2) which integrates cellular responses to metabolic and environmental stressors. As a key ion involved in energy metabolism and DNA/RNA stabilisation, magnesium has a pervasive role as modulator of many of the pathways that make up the MTR trinity. This review has indicated the complexities of how such processes might act,either independently, additively or cumulatively in normative ageing and disease.

One important sequitur from the perspective of the MTR is that the use of TL solely as a cellular biomarker of ageing to study disease will yield equivocal findings based on disease context. In keeping with this, many studies using TL as a singular biomarker of ageing have often yielded equivocal results. This is particularly pertinent to the use of TL as a biomarker in cancer studies. Given the role that telomeres play in cellular homeostasis and the multi-faceted nature of the telomere nucleo-protein complex, a more complete understanding of the various pathways that link cellular metabolism and telomere homeostasis will help resolve any such equivocation. An examination of the non-canonical functions of TERT, for example, in the context of the MTR might be of particular interest in this regard.

Another avenue that deserves further study is the impact of disruption of circadian rhythms on the ability of cells to both co-ordinate maintenance of telomere nucleo-protein complexes and regulate ROS production, which may in turn affect cellular and tissue health span and the rate of physiological ageing.
